# A Case of Anti-LGI1 Limbic Encephalitis Presenting With Non-convulsive Status Epilepticus and Requiring a Long Period of Hospitalization

**DOI:** 10.7759/cureus.78015

**Published:** 2025-01-26

**Authors:** Naotaka Miyazawa, Minatani Shinobu, Akitoshi Takeda, Yoshiaki Itoh

**Affiliations:** 1 Neurology, Graduate School of Medicine, Osaka Metropolitan University, Osaka, JPN

**Keywords:** autoimmune encephalitis, facio-brachial dystonic seizure, focal impaired awareness seizure, periodic discharge, steroid pulse therapy

## Abstract

An 81-year-old man was brought to our hospital with tonic-clonic seizures and a gradual progression of cognitive dysfunction. Four months prior, he experienced transient episodes of stiffness and unresponsiveness. One month later, the patient began to exhibit disorientation to time and place. Upon admission, fluid-attenuated inversion recovery (FLAIR) brain magnetic resonance imaging (MRI) revealed hyperintense lesions in the right limbic cortex. Electroencephalography (EEG) revealed lateralized periodic discharges in the right temporal lobe. Serum anti-LGI1 antibody was detected. Based on these findings, anti-LGI1 limbic encephalitis (LE) was diagnosed. His symptoms improved with corticosteroid treatment; however, six months of hospitalization were necessary for him to regain independence in daily living. Although the prognosis of anti-LGI1 LE is generally favorable, older age at onset, non-convulsive status epilepticus, diffuse limbic lesions, and delayed treatment may have influenced the clinical course in this case.

## Introduction

Limbic encephalitis (LE), a disorder affecting the medial temporal lobe, amygdala, and cingulate gyrus, is characterized by psychiatric symptoms, disturbances in consciousness, and convulsions [1,2]. Herpes simplex virus is the most common cause of LE, followed by autoimmune encephalitis, which is classified into two groups: one associated with classical onco-neuronal antibodies targeting intracellular antigens and the other associated with antibodies against neuronal cell surface antigens [3-6]. Generally, the latter is more responsive to immunotherapy, and prompt and enough amount of therapy should be administered. Anti-LGI1 antibody, a lately identified antibody that targets a neuronal cell surface antigen, has been shown to induce limbic encephalitis [7-9]. Here, we report a case of anti-LGI1 antibody-positive LE presenting with focal impaired awareness seizures, non-convulsive status epilepticus, and tonic-clonic seizures, alongside slowly progressive cognitive decline. The patient in the present report was successfully treated with intravenous methylprednisolone pulse therapy, followed by long-term corticosteroid therapy.

## Case presentation

An 81-year-old man was brought to the emergency room of our hospital because of tonic-clonic seizures. Four months previously, he had experienced transient episodes of limb stiffness or unresponsiveness with staring eyes and consulted a nearby doctor. Magnetic resonance imaging (MRI) of the brain revealed no abnormalities. Focal seizure with impaired awareness was suspected, and lacosamide, 100 mg b.i.d., was prescribed. One month later, the patient became frequently disoriented in terms of time and place. He would attempt to go to the workplace he had retired from long ago or fail to return home as he had forgotten the room number of his apartment. One month prior to admission, he was referred to the memory clinic at our hospital because of worsening nocturnal restlessness and early morning wandering. Neuropsychological assessment revealed a Mini-Mental State Examination (MMSE) score of six out of 30 and a Revised Hasegawa’s Dementia Scale (HDS-R) score, a comprehensive assessment of cognitive function in Japan, of four out of 30. Regular screening tests for dementia did not reveal significant findings. Lacosamide was replaced with levetiracetam, 500 mg b.i.d.; however, the patient’s cognitive function deteriorated non-specifically. Finally, the patient experienced a tonic-clonic seizure and was admitted to our hospital. The patient had a medical history of hypertension. He did not smoke or drink and had no family history of neurological disorders.

On admission, the patient’s body temperature was 35.8℃. His blood pressure was 143/86 mmHg, and his pulse rate was 64/min and regular. Percutaneous oxygen saturation was normal. No autonomic dysfunction was observed. No other abnormalities were noted on the physical examination. Neurological examination revealed unconsciousness, with a Glasgow Coma Scale score of E1V1M2, even several hours after the seizure. Neck stiffness is also observed. No cranial nerve abnormalities or involuntary movements were observed. Muscle tone and deep tendon reflexes were normal without neuromyotonia and no pathological reflexes were detected. Due to the disturbance of consciousness, sensory and coordination assessments could not be performed in detail.

Blood tests revealed no electrolyte abnormalities and a sodium level of 145 mEq/L (reference range: 135-150 mEq/L). There was no evidence of an inflammatory response. Tests for autoantibodies associated with collagen diseases (antinuclear antibody, anti-DNA antibody, antibodies against MPO-ANCA, PR3-ANCA, SSA, or SSB), anti-glutamic acid decarboxylase antibody, or anti-aquaporin 4 antibody were negative. Finally, a serum test confirmed the presence of anti-LGI1 antibodies.

Cerebrospinal fluid (CSF) examination revealed pleocytosis (29/μL (<5/μL), mononuclear cells 100%), an elevated protein level (97 mg/dL (<45 mg/dL)), a normal glucose level, and no oligoclonal bands. The immunoglobulin G index increased to 0.89 (<0.75). A multiplex polymerase chain reaction panel test, including that for herpes simplex virus, yielded negative results.

**Figure 1:**
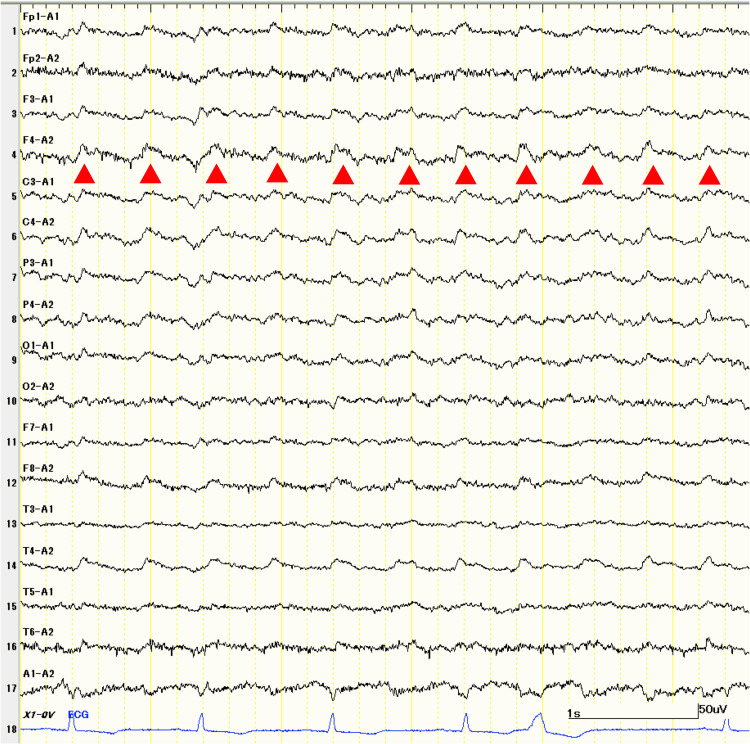
EEG recorded about a month before admission. Periodic discharges (arrowheads) are observed diffusely in the right frontotemporal areas (F4, T4). The amplitude of background activity in the occipital lobe is reduced. EEG, electroencephalography

Electroencephalography (EEG) performed one month before admission showed lateralized periodic discharges in the right frontotemporal area (Figure 1).

These discharges were smaller in amplitude and more diffusely spread beyond the right temporal lobe compared to common LE such as herpes encephalitis. The amplitude of the background activity in the occipital lobe was also reduced compared to that in typical cases of LE. Periodic discharge was no longer observed soon after the initiation of treatment.

**Figure 2:**
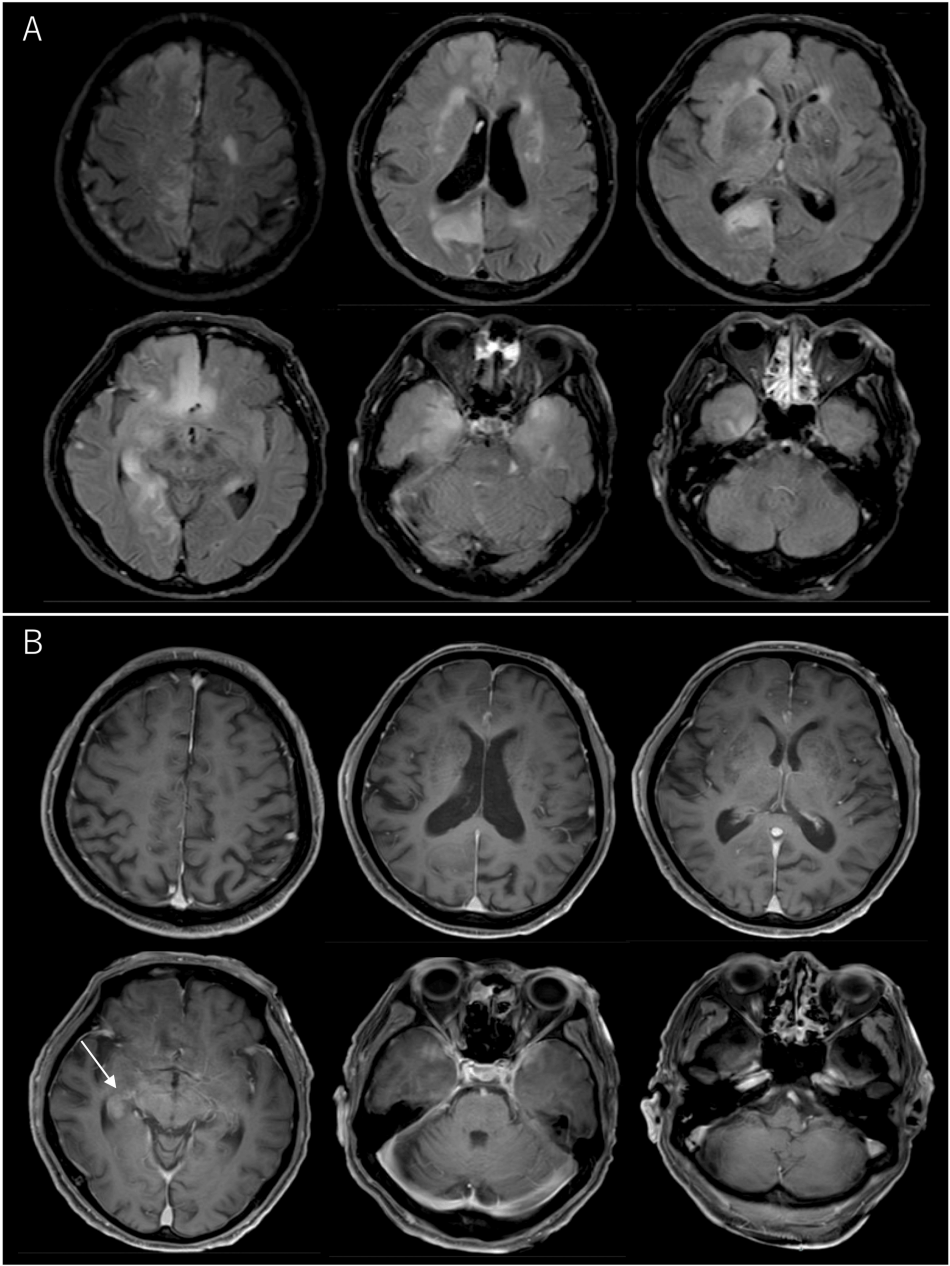
Brain MRI on the day of admission. A: FLAIR images reveal hyperintensity in the right limbic system, including the medial temporal, frontal, parietal, and occipital lobes, as well as in the left medial temporal lobe. B: Contrast-enhanced MRI shows hyperintensity in the right temporal lobe (arrow). MRI, magnetic resonance imaging; FLAIR, fluid-attenuated inversion recovery

Brain MRI on the day of admission revealed hyperintensities in the right limbic system, including the medial temporal lobe, amygdala, and cingulate gyrus, as well as in the left medial temporal lobe, on T2-weighted images (T2WI) and fluid-attenuated inversion recovery (FLAIR) images (Figure 2).

**Figure 3:**
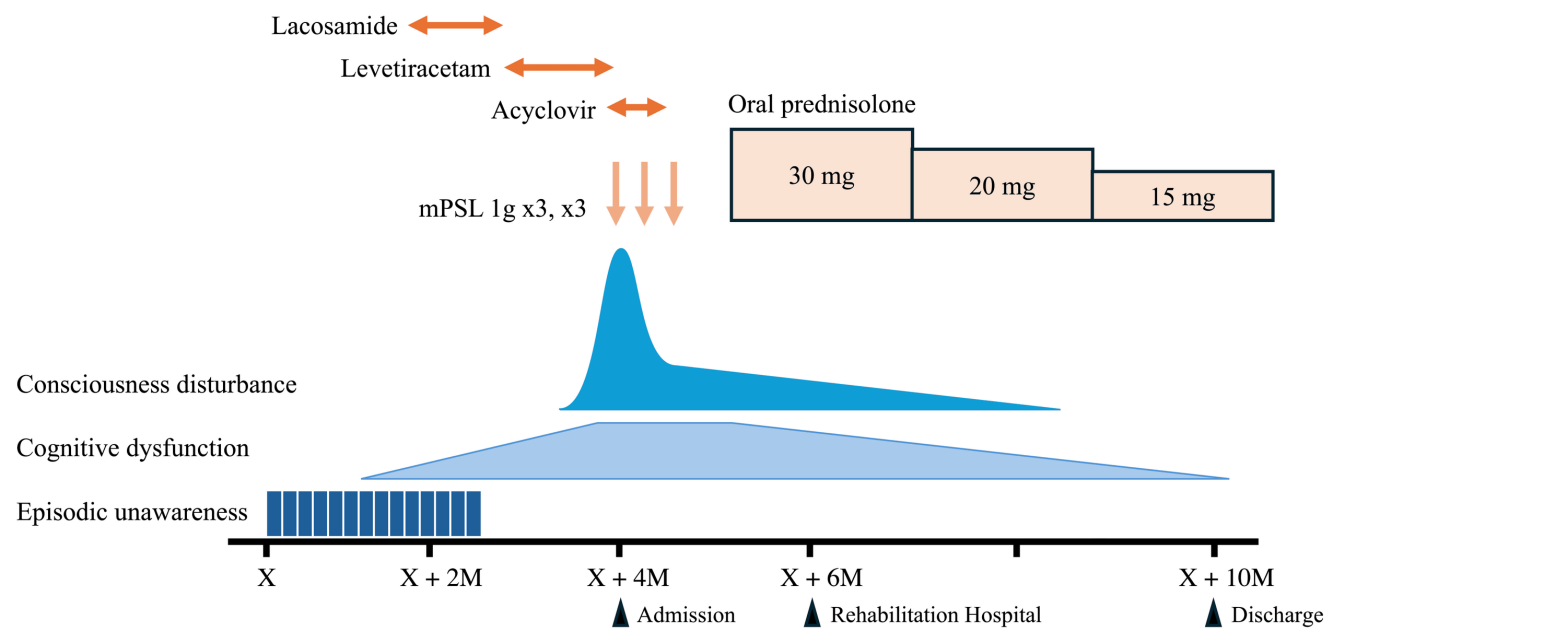
Time course of clinical manifestations and treatments. The patient responded quickly to intravenous mPSL pulse therapy but recovered slowly thereafter. X: onset, M: month; mPSL, methylprednisolone

Contrast-enhanced MRI revealed hyperintensity in the right temporal lobe. Contrast-enhanced computed tomography of the chest and abdomen revealed no evidence of tumors. Based on these findings, the patient was diagnosed with anti-LGI1 LE. On admission, steroid pulse therapy and intravenous acyclovir (10 mg/kg for every eight hours) were initiated because herpes encephalitis could not be excluded (Figure 3).

The patient received three courses of steroid pulse therapy, each consisting of intravenous methylprednisolone of 1 g for three consecutive days, followed by oral prednisolone. The result of positive LGI1 antibodies was noticed during the treatment. His consciousness improved rapidly and he regained orientation. The patient was transferred to another hospital for further treatment. Six months later, the patient recovered independence in activities of daily living (modified Rankin Scale (mRS) 1) and was discharged.

## Discussion

The present case was diagnosed as anti-LGI1 LE, with possible facio-brachial dystonic seizures, epileptic seizures, and slowly progressive cognitive decline. Focal impaired awareness seizures and memory disturbances suggested temporal lobe involvement, as confirmed by EEG and MRI findings (Figure 1 and Figure 2). On the basis of the clinical course and CSF results, LE was suspected upon admission. Symptoms of LE typically include memory impairment, epileptic seizures, and psychiatric abnormalities [4]. The present case exhibited these characteristic features, although the progression over four months was relatively slow.

The differential diagnosis of LE includes infectious, autoimmune, demyelinating, metabolic, nutritional, toxic, and neoplastic diseases and connective tissue diseases [4]. Based on the detection of the LGI1 antibody, the patient was diagnosed with an anti-LGI1 LE.

Anti-LGI1 LE was initially reported to be associated with antibodies against the voltage-gated potassium channel complex (VGKC) [9-11]. More recently, LGI1 has been identified as a specific target antigen in cases previously attributed to potassium channel antibodies [8,12]. A meta-analysis of 139 patients with anti-LGI1 LE in Asia reported a mean age of 54.17 years, with 61.2% male patients. Tumor associations were rare (3.3%) [13]. However, the present case occurred at a significantly older age, which may explain the delay in seeking evaluation despite transient unresponsiveness and mild cognitive decline.

Clinical features of anti-LGI1 LE include various types of seizures, slow progressive cognitive decline, and psychiatric abnormalities [8,13,14]. In this case, focal impaired awareness seizures and tonic-clonic seizures, both common in anti-LGI1 LE [13], were observed. Additionally, facio-brachial dystonic seizures, characterized by brief dystonic movements affecting the ipsilateral face and arm and specific for anti-LGI1 LE [15], may have been present as transient stiffness in this patient, although detailed descriptions of these episodes are unavailable.

Hyponatremia, a hallmark of anti-LGI1 LE observed in 56.3% of cases [13], was absent in this patient despite repeated blood tests. Anti-LGI1 antibodies were detected in 82.5% of CSF samples and 93.6% of serum samples from patients with anti-LGI1 LE [13]. In the present case, an antibody was detected in the serum. Dual antibody positivity for neuronal cell surface antigens is exceedingly rare [16] and was not observed in this case. CSF abnormalities were reported in 33.3% of patients with anti-LGI1 LE, including pleocytosis in 10.3% and elevated protein levels in 21.4% [13]. Pleocytosis and increased protein concentrations were observed in this patient.

EEG abnormalities are observed in approximately 80% of anti-LGI1 LE cases [13,17]. In a systematic review, disturbance of the background rhythm was observed in 46.6% of cases, and epileptiform discharges, including spikes, sharp waves, and slow waves, were observed in 57.3% of cases [17]. The lateralized periodic discharges observed in this case have been previously reported in only one of 151 cases of anti-LGI1 LE [17,18], suggesting that the patient was in the ictal-interictal continuum of non-convulsive status epilepticus [19,20]. To assist in evaluating autoimmune encephalitis in patients with epilepsy, the Antibody-Prevalence-in-Epilepsy-and-Encephalopathy (APE²) and antibodies contributing to focal epilepsy signs and symptoms (ACES) scoring systems incorporate clinical features, CSF findings, and MR results. Based on these features, this patient’s scores were above the cutoff for both APE2 (score 5, cutoff ≥4) and ACES (score 4, cutoff ≥2).

MRI abnormalities have been reported in 54.1% of patients with anti-LGI1 LE [13]. The lesions typically involved the medial temporal lobe (26.1%), the hippocampus (20.7%), and the basal ganglia (10.8%) [13]. In this patient, diffuse hyperintensity was observed in the medial cortex of the right hemisphere, including the frontal, temporal, parietal, and occipital cortices. Most of the insular cortex and parts of the adjacent basal ganglia also appeared hyperintense on FLAIR imaging. Such large lesions covering the whole limbic system have been reported in only a few patients with anti-LGI1 LE [13] and might be associated with poor outcomes. In patients with herpes simplex encephalitis, extensive lesions involving three brain lobes that are significantly associated with poor outcomes are rarely reported.

First-line treatment for anti-LGI1 LE includes corticosteroids alone (40.7%), intravenous immunoglobulin (IVIG) alone (5.2%), or a combination of both (48.9%) [13]. Immunosuppressants were used in only 3% of the cases [13]. The prognosis of anti-LGI1 LE is generally favorable, with a mRS of 0-2 in 91.7%, 3-4 in 3.3%, 5 in 0.8%, and 6 in 2.5% [13]. The mean duration of hospitalization was 27.86 days [13] and relapse occurred in 22.8% of cases [13]. In the present case, methylprednisolone pulse therapy was followed by oral prednisolone, which led to a clear response. No additional IVIG was administered. However, the patient required six months of hospitalization to recover to an mRS score of 2, which is significantly longer than the average duration reported in previous studies.

## Conclusions

Here, we present a rare and unusually severe case of anti-LGI1 LE characterized by late-onset and disabling symptoms, including an ictal-interictal continuum of non-convulsive status epilepticus. Detailed examinations revealed rare findings such as lateralized periodic discharges on EEG and diffuse involvement of the limbic system on MRI. The patient required prolonged hospitalization for recovery. Advanced age, status epilepticus, and extensive lesions may have contributed to the severity in this case.

## References

[1] Brierley JB, Corsellis JAN, Hierons R, Nevin S (1960). Subacute encephalitis of later adult life. mainly affecting the limbic areas. Brain.

[2] Corsellis JA, Goldberg GJ, Norton AR (1968). "Limbic encephalitis" and its association with carcinoma. Brain.

[3] Graus F, Delattre JY, Antoine JC (2004). Recommended diagnostic criteria for paraneoplastic neurological syndromes. J Neurol Neurosurg Psychiatry.

[4] Asztely F, Kumlien E (2012). The diagnosis and treatment of limbic encephalitis. Acta Neurol Scand.

[5] Dalmau J, Tüzün E, Wu HY (2007). Paraneoplastic anti-N-methyl-D-aspartate receptor encephalitis associated with ovarian teratoma. Ann Neurol.

[6] Graus F, Titulaer MJ, Balu R (2016). A clinical approach to diagnosis of autoimmune encephalitis. Lancet Neurol.

[7] Marquetand J, van Lessen M, Bender B, Reimold M, Elsen G, Stoecker W, Synofzik M (2016). Slowly progressive LGI1 encephalitis with isolated late-onset cognitive dysfunction: a treatable mimic of Alzheimer's disease. Eur J Neurol.

[8] Lai M, Huijbers MG, Lancaster E (2010). Investigation of LGI1 as the antigen in limbic encephalitis previously attributed to potassium channels: a case series. Lancet Neurol.

[9] Buckley C, Oger J, Clover L, Tüzün E, Carpenter K, Jackson M, Vincent A (2001). Potassium channel antibodies in two patients with reversible limbic encephalitis. Ann Neurol.

[10] Thieben MJ, Lennon VA, Boeve BF, Aksamit AJ, Keegan M, Vernino S (2004). Potentially reversible autoimmune limbic encephalitis with neuronal potassium channel antibody. Neurology.

[11] Vincent A, Buckley C, Schott JM (2004). Potassium channel antibody-associated encephalopathy: a potentially immunotherapy-responsive form of limbic encephalitis. Brain.

[12] Irani SR, Alexander S, Waters P (2010). Antibodies to Kv1 potassium channel-complex proteins leucine-rich, glioma inactivated 1 protein and contactin-associated protein-2 in limbic encephalitis, Morvan's syndrome and acquired neuromyotonia. Brain.

[13] Ghimire P, Khanal UP, Gajurel BP (2020). Anti-LGI1, anti-GABABR, and Anti-CASPR2 encephalitides in Asia: a systematic review. Brain Behav.

[14] Yang X, Li AN, Zhao XH, Liu XW, Wang SJ (2019). Clinical features of patients with anti-leucine-rich glioma inactivated-1 protein associated encephalitis: a Chinese case series. Int J Neurosci.

[15] Irani SR, Michell AW, Lang B (2011). Faciobrachial dystonic seizures precede Lgi1 antibody limbic encephalitis. Ann Neurol.

[16] Nakano T, Chihara N, Matoba K (2022). Immunotherapy-responsive Non-paraneoplastic encephalitis with antibodies against GAD, LGI1, and GABA(A) receptor. Intern Med.

[17] Roberto KT, Espiritu AI, Fernandez ML, Gutierrez JC (2020). Electroencephalographic findings in antileucine-rich glioma-inactivated 1 (LGI1) autoimmune encephalitis: a systematic review. Epilepsy Behav.

[18] Shin YW, Lee ST, Shin JW (2013). VGKC-complex/LGI1-antibody encephalitis: clinical manifestations and response to immunotherapy. J Neuroimmunol.

[19] Masuda T, Kimura N, Nakamura K, Okazaki T, Arakawa R, Kumamoto T (2011). A case of limbic encephalitis repeated aphasic status epilepticus with periodic lateralized epileptiform discharges [Japanese]. Rinsho Shinkeigaku.

[20] Kaplan PW, Rossetti AO, Kaplan EH, Wieser HG (2012). Proposition: limbic encephalitis may represent limbic status epilepticus. A review of clinical and EEG characteristics. Epilepsy Behav.

